# The Foreign Journals: Pathology, Practical Medicine, and Therapeutics

**Published:** 1839-10

**Authors:** 


### PATHOLOGY, PRACTICAL MEDICINE, AND THERAPEUTICS.

### *Case of Apoplexia Cutanea.* By Dr. Otto, of Copenhagen.

I have seen and treated a case of this disease which, I must confess, I should not have known, unless I had read the observations and remarks on the subject by Dr. Léveillé, in the *Revue Médicale.* I was one morning early called to a carrier, who, the evening before, had gone to bed half drunk, and on awaking in the morning found a great part of his body—the breast, the back, and the upper extremities, covered by widely-spread, dark-blue ecchymoses, not, however, elevated above the level of the skin. He and his wife suspected that he had had during the night an epileptic attack, and had during it so beaten himself as to cause the blue spots; but as such spots do not usually occur so soon after blows and falls, and as the usual yellow colour on the places which have suffered long from blows was nowhere to be seen, and especially as the patient had never been affected with epilepsy before, I could not agree in the opinion. Although he was so far in his senses as to answer in an ordinary way my questions, and to express his fear of the blue spots, still he was sometimes quite stupid, and suffered from *tremor artium*, which, however, was not surprising to me, as I knew him to be a confirmed toper. His face was not red, his eyes gave no indication of congestion towards the head, and he affirmed that he had neither headach nor vertigo. There was no paralysis or any other symptom indicative of apoplexy. I had therefore every reason to consider the disease as a true *apoplexia cutanea*, of the kind called by Léveillé *apoplexia cutanea ecchymatica*, to distinguish it from *apoplexia cutanea exanthematica*, which is characterized by large irregular spots elevated above the surface of the skin; in the last-mentioned form of the disease, the blood in its way to the skin is still in the arterial capillaries; in the first-mentioned form, as in the present case, the blood is effused into the subcutaneous cellular tissue. The pulse was full and quick, the tongue clean, the skin warm, and the bowels open.

I took blood from the patient, ordered rest, a regulated diet, sulphate of magnesia dissolved in water, and to bathe the ecchymosis with a saturnine lotion. The same day the man ceased to speak incoherently. No other symptom occurred; the spots gradually became paler, but did not completely disappear until three weeks. He has had no relapse. *Casper's Wochenscrift. May,* 1839.

---

### *Case of Chronic Farcy terminating in Acute Glanders.* By M. Lenepveu.

A. P. æt. fifty, groom, admitted to the Hospital St. Louis, under M. Jobert, 8th Sept., 1838; has been employed for eight years in an establishment for hackney carriages; a number of the horses had been affected with glanders and farcy during the course of the year. When admitted, he had been four months ill; his complaints having commenced with wandering pains in the shoulders, loins, and limbs, without fever, followed, about a month after, by swelling of the left ankle-joint; almost at the same time several soft tumours, free from redness or pain even on pressure, appeared in the limbs, the latter being elsewhere free from tumefaction. These tumours were, *on the patient's admission*, found to be subcutaneous abscesses, unattended with engorgement either of the veins, lymphatics, or neighbouring tissues; inguinal glands slightly swollen but indolent; no fever, appetite good, general state satisfactory; no traces of syphilis discoverable either on the skin, in the pharynx, or nares. The patient was put on a course of tonics; the tumours to be rubbed with an ioduretted ointment.—*Sept. and October.* Tumours slightly diminished in size; two on the foot and right leg have opened spontaneously and continue to discharge—the former a serous yellow fluid in small quantity, the latter grayish dusky-coloured pus in much greater abundance, gradually changing to a blackish tint, and mingled with shreds of gangrenous cellular tissue.—*November.* The patient is still without fever and has a good appetite, but has lost flesh considerably.—*December.* He grows morose and low-spirited; has lost appetite and sleep; decumbiture constantly dorsal; movements difficult; pain in head, limbs, and trunk; tumours now exceedingly tender; the cicatrix adjoining the left eye has inflamed, and furnished for some days past a good deal of yellow pus; cough, and white ropy expectoration, lasting a few days only. A fit of shivering now came on, followed by fever; which never disappeared till the patient's death; stupor without delirium; extreme emaciation; involuntary stools without diarrhœa or gastric symptoms; tongue natural.—25*th.* Abscesses of the thighs and arms, apparently absorbed; fistula of the left foot changed into an ulcer, with grayish fundus and livid perpendicular borders.—28*th.* Erysipelatous redness of forehead extending to the scalp; violent nocturnal delirium, prostration by day.—29*th.* Livid spots over each eyebrow; white discharge from surface of right eye; under frontal ecchymosis are small flat soft tubercles; vesicles on the eyelids; dark-coloured ulcer over right frontal eminence; violet tubercular elevation at the root of the nose; the erysipelatous tinge is changed into a blueish yellow hue. Similar ecchymotic appearance on shoulder, with serous phlyctenæ; on right thigh three isolated white pustules, surrounded with a small red circle, resembling in form those of modified smallpox: the redness of the skin, ecchymosis, vesicles, and pustules, were all developed in about twenty-four hours. The patient expired at three P.M. —*Dissection forty-two hours after death.* Inguinal glands swollen and red, with a small white nucleus in the centre; under the skin of the thighs, in the situation of the tumours, is some matter of dirty white colour, concrete, soft, and not surrounded with a pseudo-membrane; similar matter on surface of left deltoid muscle, and small purulent collections in that of the right side, pus in the left ankle-joint, other articulations healthy, bones of the limbs normal, muscles of right leg infiltrated with purulent sanies.—*Head.* Brain healthy; meningeal vessels and sinuses gorged with blood.—*Nares.* Pituitary membrane of left nostril healthy; that of right side injected and studded with pustules, extending from the inferior turbinated bone to the Eustachian tube; the membrane appears ulcerated in several points after the removal of the thick yellow opaque matter deposited on its surface; within the group of pustules the mucous membrane is swollen, of violet colour, and speckled with whitish points. The injection extends into the frontal and sphenoid sinuses; the lining membrane of the latter contains a small clot of blood in its substance.—*Larynx.* Small deep ulceration above the right corda vocalis; a very minute cartilaginous-like patch between the laminæ of the corresponding arytæno-epiglottic ligament.—*Lungs.* Inferior lobes engorged; numerous points of lobular pneumonia, varying in size from that of a millet-seed to that of a filbert, the centre generally formed of a yellow concrete matter, in some instances containing pus, and surrounded with dark and hardened pulmonary tissue; slight redness of ultimate bronchi.—*Heart and pericardium* healthy.—*Liver* contains some fibrinous matter, such as that described in the lungs.—*Spleen* much enlarged, firm and denser than natural, and of reddish colour; contains several small clots of blood and a number of large, yellow, fibrinous, dense, and resisting masses; a layer of similar matter underneath the fibrous capsule.—*Stomach and intestines* healthy throughout.—*Mesenteric glands* enlarged.

[We refrain, until the publication of M. Rayer's promised work on Chronic Farcy in the Human Subject, from commenting on this remarkable case; the post-mortem examination was made in the presence of several hospital physicians, and the diseased parts shown to the Members of the Royal Academy of Medicine.]

*L'Expérience. Janvier,* 1839.

*On Cod-liver Oil and Ray-liver Oil in Scrofulous Affections.* By M. Gouzée.

This oil, formerly recommended by Dr. Percival and many German physicians, has lately been employed in France and Belgium. M. Gouzée declares it to be one of those rare medicines which produce marvellous results; that it is useful in scrofula,—above all in that form producing osseous alterations in young subjects; and even in chronic articular rheumatism, &c.

Case i. About ten years ago, M. G. was consulted regarding the health of a child afflicted with rickets, having a pigeon-breast and a dorsal curvature. The child had constant fever, acute pain in the precordial region, with great impulsive action of the heart, laborious breathing, sleepless nights, constant reclination upon the left side, constant diarrhœa, and irritability of the stomach. It had been in this state some time, and was gradually sinking. The oil of the liver of the ray (*Raia pastinaca*) was administered in the dose of half a teaspoonful morning and evening, and gradually increased to half a tablespoonful. The fever rapidly diminished, the precordial pains disappeared, the diarrhœa ceased, and in less than a month, the little patient was walking about. The remedy was continued for several months, and at the present time, nothing remains but a slight narrowing of the chest, and a trifling projection of the head forwards, the result of the dorsal curvature.

Case ii. A child four years old, had an enlarged head, red and swollen countenance, large abdomen, thin and feeble extremities, especially the inferior. The oil was given in the same doses, and in two months the infant walked; the abdomen assumed its usual normal size, and the enlargement of the head was the only remaining trace of the disease.

Case iii. A little girl, ten years of age, had been suffering for three years from a scrofulous enlargement of the left elbow, for which all the usual remedies had been unsuccessfully administered. The integuments were white and shining, the joint was completely anchylosed at right angles, there were acute pains, and for the last twelvemonth, a large fungous ulcer occupied the surface. There was considerable emaciation, violent pains in the abdomen, constipation, and loss of appetite. Local emollient baths were ordered, and ferruginous preparations given internally without success. The oil was administered in the usual doses, and after a month, the general health was reestablished, the pains in the joint had ceased, the swelling was less, and the ulcer had contracted and lost its unhealthy appearance. In two months the patient could move the elbow with facility, the joint had almost regained its normal size, and nothing remained of the former disease but a slight swelling and a want of power to perform perfect extension.

The fish is taken in the summer, and the oil is extracted only from the liver, which is sold at Antwerp. In commerce, there is an impure oil met with, extracted from the livers of various other fishes, and called in Flanders *lever traen;* this is not possessed of the therapeutical power of the former. M. Van Camp, with Hopfer and Hausmaus, have discovered the existence of iodine in this oil. It has a brownish yellow colour, and an insipid taste which leaves in the mouth a particularly disagreeable fishy odour; however, patients, even infants, take it without difficulty, especially after the first doses. M. G. prescribes it, without any adjuvant, morning and evening, a teaspoonful for young children, and a tablespoonful for adults, gradually increasing the dose till it is doubled.

*Bulletin Gènèral de Thèrapeutique. Mai,* 1838.

---

*On Fatty Disease of the Liver* (*Pimelosis Hepatica.*) By Dr. Albers, of Bonn.

The fatty disease of the liver has of late been sometimes regarded as hypertrophy of that organ, sometimes as cirrhosis, and sometimes as the result of chronic inflammation. But it is a peculiar affection, and perfectly distinct from all these: it differs from hypertrophy in the alteration which the tissue undergoes; from cirrhosis, by the lobules being unaltered or but slightly atrophied; and from chronic inflammation, by the absence of all symptoms of such an affection even when the disease is completely developed.

The liver when affected with this disease is always large, and surpasses the normal size; the increase commonly affects the whole organ, but especially its basis and the right side; and the more advanced the disease, the greater is the enlargement. The colour of the liver is externally reddish yellow, or marbled with shades of yellow; internally, it is yellow shaded with red in the early, and golden yellow in the later periods of the disease. The substance is firm and compact like that of the liver of a phthisical patient, and it retains its firmness for a day or two after its removal from the body. Its weight is increased. Its vessels are all permeable and may be traced to the minute parts; the branches of the vena portæ are all very large; the blood which they contain is black and greasy. The hepatic artery and ducts appear healthy; the bile is very dark and fluid.

If a portion of a liver, thus golden-coloured, be examined with the microscope, it appears like a pale white spongy substance, containing separate cells of a bright colour, with a delicate fluid, and here and there some dark brown points the acini of the liver, which are smaller than in their normal state. Thus, while the tissue between the lobules is abundant and hypertrophied, the lobules themselves are atrophied; and, in all stages of the disease, the increase of the one is proportioned to the decrease of the other. It is in this hypertrophied interlobular cellular tissue of the liver, that the deposition of fat chiefly takes place.

In general, the fatty disease of the liver appears in that organ alone, and all the other parts of the body are atrophied; enlargement and accumulation of fat in the liver being generally connected with an extreme condition of emaciation. In the late French works on pathological anatomy, it is said that the fatty condition of the liver is very common in tuberculous affections of the lungs. But the liver naturally contains a remarkable quantity of fat, which is sometimes increased in many diseases, as well as in phthisis, without constituting what can fairly be called fatty disease of the liver; for the organ retains its brownish colour, the lobules remain normal, and the cellular tissue is only a little increased in quantity. The surface of a section of such a liver is not yellow, but a mixture of brownish yellow and gray; and much of the enlargement which it presents, depends on the great accumulation of blood in the vessels.

With respect to the symptoms of this disease, it commences with all the manifest signs of disordered digestion, pain, and weight in the epigastrium and right hypochondrium, occasional vomiting, and entire loss of appetite. At a later period, there are prominence and tension of the hypochondrium, the pain being increased by pressure, but often relieved by the abstraction of blood. The skin becomes pale yellow, but has the hue rather of that which is seen in organic disease of the stomach, than of common jaundice. As the disease advances, the colour of the skin increases in intensity, and the greatest emaciation takes place; but if the disease be not complicated, dropsy never occurs, though the emaciation is extreme. It continues for from four to fifteen months, till at the last, difficulty of breathing and great weakness supervene, and the patient dies gradually exhausted.

The most important diagnostic is the great emaciation without dropsy; for in hypertrophy, cirrhosis, and all other chronic diseases of the liver, the latter always occurs, both in the form of ascites and of anasarca. The pain relieved by bleeding is also an important sign, being more rarely and less distinctly observed in hypertrophy or cirrhosis.

As yet no remedy is known for this affection; and the immediate cause of the accumulation of fat is equally uncertain. It affects persons of upwards of thirty-five years of age, and both sexes equally.

*Rust's Magazin für die gesammte Heilkunde.* 1839.

---

*On the Medical Use of Alum, in Diseases of the Heart.* By Dr. SCHLESIER.

IN this short paper, Dr. Schlesier calls attention to the great benefit derived from the internal use of alum in dyspepsia, and also and especially in dilatation of the heart. He quotes the case of a girl, aged 10, whose heart was so much dilated, that her case had been considered hopeless; but whom he succeeded in curing by the prolonged exhibition of alum. The stroke of the heart was perceptible over all the left side to the fourth false rib, and its motion appeared to the eye in the intercostal spaces like a rolling wave. The sternum and ribs of the left side were drawn upwards; the respiration was short and hurried, and accompanied by a short, dry cough, and considerable dyspnœa; the pulse was about 100, soft and small, and the child was emaciated and pale. In this state alum was ordered, in combination with rhatany and digitalis, and its effects were truly wonderful. The apex of the heart became weekly more elevated, and the stroke of the heart more limited in extent, whilst the general condition of the patient improved so much that in four weeks she could be removed home. The treatment was continued for a considerable time with great benefit; but still a slight degree of dilatation remained, and the heart was easily affected by exercise and mental emotions.

[The auscultatory diagnosis of the disease ought to have been given, as also the dose of the remedy.] *Medicinische Zeitung*, No. xlvii. 1838.

---

*On Arteritis.* By E. Bonetti.

The author of this paper endeavours to establish the reality of inflammation of the internal tunic of arteries in opposition to the doctrine strenuously maintained by Rasori, of the physiological impossibility of such lesion.* He believes he has succeeded, grounding his belief on the following results, obtained by observing the effects of the introduction of croton oil into the arteries of rabbits, sheep, and horses. 1st. The internal tunic of the arteries possesses sensibility. 2d. It contains capillary vessels, which serve as the nidus of the inflammatory process. 3dly. The same phenomena are observed to take place in the internal tunic of arteries, as in every other system endowed with nerves and blood-vessels, namely, the successive appearance, under the action of a stimulus, of irritation, congestion, and inflammation. 4thly. Inflammation of this membrane comes rapidly to an issue, which consists in ulceration and destruction of tissue, by suppuration and sphacelus.

[We have made this extract simply to show the state of opinion in Italy, on a very interesting question, and need hardly remind the reader that the accurate researches of the French school have proved that truth lies between the opposing and exclusive dogmas of Frank and Rasori. M. Bonetti will not, we trust, in the ardour of discovery, arrive at the adoption of M. Bouillaud's whimsical absurdity, that "*fever is an angeio-carditis, of more or less intensity*."† The value of proposition 4 may be tested by the results of Bizot, noticed in a recent number.‡]

*Giornale delle Scienze Med.-Chir.*, No. xxxviii. *Agosto*, 1837.

---

*Clinical Experiments on the Emetic and Sudorific Properties of the Hydrated Sulphuret of Antimony, with excess of Sulphur.* (Berzelius.) By M. A. Toulmouche, M.D.

From a series of experiments, of which the individual results are given in a Table, M. Toulmouche draws the following conclusions on the properties of this salt.

1. This preparation of antimony acts with greater certainty as an emetic, when given in doses of one or two grains, than when exhibited in larger quantities.
2. It induces vomiting in smaller doses than kermes.
3. Its emetic action, although less uncertain than that of kermes, is still far from sure, inasmuch as it is only developed in somewhat more than half the cases of its administration.
4. It acts much less frequently as a purgative than as an emetic; whereas the contrary is true of kermes.

* Rasori, Teoria della Flogosi, p. 250 et seq.

† Art. Fièvre, du Dict. de Méd. et de Chir. Prat. tom. viii. p. 86.

‡ Brit. and For. Med. Rev. vol. VI. p. 45.

5. Like the last-named compound, it may be given with impunity in large doses, and in other affections besides rheumatism and pneumonia; in these cases its emetic and purgative effects appear to diminish in proportion to the increase of the dose.

6. The sudorific properties attributed to it by writers on materia medica are by no means incontestable; in 102 cases of its exhibition it acted on the skin thirteen times only. *Gazette Médicale de Paris*, No. xiv. *Avril*, 1839.

---

*A Contribution to Psychical Anthropology.* By Dr. Malin, of Lübbenau.

We have read, we think, in Dr. Prichard, the case of one of the members of a very stupid family, who was himself formerly as stupid as the rest, receiving an injury of the head, and after that manifesting more than ordinary intellectual endowments. Dr. Malin relates under the above title, in Casper's Wochenschrift, three similar cases, and concludes his communication thus:

"What a benefit to humanity, if it could be determined under what condition, and in what cases—at what place and with what force such a blow or fall would be of use! At any rate, I can adduce in favour of my assertion the experience of a very intelligent veterinary surgeon, who saw a horse affected with the staggers (Dummkoller) radically cured, after it had received from its master a heavy blow on the head with a hammer, with the intention of killing it."

*Casper's Wochenschrift.* *May* 11, 1839.

